# Thermal Radiative Copper Oxide Layer for Enhancing Heat Dissipation of Metal Surface

**DOI:** 10.3390/nano11112819

**Published:** 2021-10-24

**Authors:** Junghyun Park, Donghyun Kim, Hyunsik Kim, Junghoon Lee, Wonsub Chung

**Affiliations:** 1 Department of Materials Science and Engineering, Pusan National University, Busan 46241, Korea; wythe017@kicet.re.kr; 2 Analysis Technical Center, Korea Institute of Ceramic Engineering and Technology, Jinju 52851, Korea; dhkim1208@kicet.re.kr (D.K.); hyunkim@kicet.re.kr (H.K.); 3 Department of Metallurgical Engineering, Pukyong National University, Busan 48513, Korea

**Keywords:** heat dissipation, electrodeposition, emissivity, copper oxide, radiative heat transfer

## Abstract

The heat dissipation of a metal heat sink for passive cooling can be enhanced by surface modifications to increase its thermal emissivity, which is reflected by a darker surface appearance. In this study, copper electrodeposition followed by heat treatment was applied to a copper substrate. The heat treatment formed a nanoporous oxide layer containing CuO and Cu_2_O, which has a dark blackish color and therefore increased the thermal emissivity of the surface. The heat dissipation performance was evaluated using the sample as a heat sink for an LED module. The surface-treated copper heat sink with a high thermal emissivity oxide layer enhanced the heat dissipation of the LED module and allowed it to be operated at a lower temperature. With an increase in the heat treatment, the thermal emissivity increases to 0.865, but the thermal diffusivity is lower than the copper substrate by ~12%. These results indicate that the oxide layer is a thermal barrier for heat transfer, thus optimization between the oxide thickness and thermal emissivity is required by evaluating heat dissipation performance in operating conditions. In this study, an oxide layer with an emissivity of 0.857 and ~5% lower thermal diffusivity than the copper substrate showed the lowest LED operating temperature.

## 1. Introduction

Thermal (or heat) management is essential for stable performance and lifetimes in various engineering systems. Therefore, the design of an engineering system requires consideration of its heat dissipation, which refers to the release of heat generated during the operation of the system to the external environment. In addition, thermal management with engineering systems and materials is important in collecting solar energy and its conversion [1,2,3]. Similar to various systems that generate heat, heat dissipation is a critical issue affecting the performance of integrated light-emitting diode (LED) systems [4,5,6,7,8]. Various technologies have been applied to improve the heat dissipation performance of engineering systems [9,10,11,12,13,14,15]. Forced convection using fans is the most widely utilized form of active cooling. Extensive experimental and simulation studies have been conducted on this form of active cooling. Moreover, various functionalized materials, including phase change material, nanocomposites, and nanocarbon materials (e.g., graphene, graphene oxide, and carbon nanotubes) have been explored for thermal management applications [16,17,18,19]. In addition, complicated cooling units that circulate cold fluids have been used for the most active form of heat dissipation [20]. Even though such active cooling systems achieve effective heat dissipation, additional energy is required for their operation. In contrast, passive cooling does not require additional energy because natural convection and thermal radiation are used to release heat to the external ambient. Although the cooling performance of passive cooling is much lower than that of active cooling, passive systems are important in mobile systems that cannot include active cooling systems (i.e., fan or liquid circulation) [21]. To enhance the heat dissipation in passive cooling, the shape and surface of the heat sink were investigated [22].

The heat dissipation in passive cooling can be improved by maximizing the thermal conductivity and optimizing the shape and geometry of the heat sink. Despite the high thermal conductivities of copper and aluminum, which are the most common metals used for heat sinks, their surfaces have a low emissivity (ε = ~0.2), which affects the thermal radiation [23]. Enhancing the surface emissivity of the heat sink is another strategy to improve heat dissipation in passive cooling systems. In aluminum, the heat dissipation can be improved by more than 4-fold by modifying the surface emissivity from 0.2 (metallic surface) to 0.8 (anodically oxidized surface) [24,25]. However, such anodic oxidation creating nanoporous thin oxide layers impregnated with black material is a unique technique for aluminum and its alloys, thus it cannot be applied to copper and other materials (e.g., steels).

One of the most effective strategies to form a thermally functional layer is a composite with thermally conductive nano/microsized particles dispersed in a polymer matrix, and thus various materials with high thermal conductivity have been explored to solve issues in heat management [26,27,28]. Among these materials, considering price and mass production, copper oxide (CuO), which is black and has a high emissivity of more than 0.9, is a promising material for modifying the metal heat sink surface to improve the heat dissipation in passive cooling. Therefore, a coating with copper oxide composite bonded with polymer resin was applied to an aluminum heat sink for high-power LED systems, where passive cooling is more desirable for energy saving and system limitation in the light-emitting direction. Then, a significantly reduced operating temperature of LED chips and enhanced heat dissipation were reported [29,30]. However, despite the high emissivity of copper oxide, its composite with polymer binder resin has a low thermal conductivity, so the heat dissipation of the coating surface can be compromised by the thickness of the coating. Moreover, the copper oxide composite (i.e., a mixture of polymer resin and ceramic particles) applied by spraying or dipping is not suitable for heat sinks made of aluminum or copper alloys with complex shapes. In addition, a polymer/ceramic composite coating creates significant interfacial thermal resistance on the metal surface because the contact between the coating layer and metal surface depends on mechanical interlocking, bonding that is not at the atomic or molecular level, showing low interfacial thermal resistance [31,32,33,34,35].

Electrodeposition is one of the most useful processes for creating copper layers on metal heat sinks with complex shapes. The surface morphology can be easily controlled through the processing conditions, which include the chemical composition, applied current, additives, and temperature [36,37,38,39]. Copper surfaces can be oxidized at high temperatures to form copper oxide showing high emissivity with enhanced surface heat dissipation. In this study, with the aim to overcome the limitations of copper oxide composite coatings, we employed copper electrodeposition and heat treatment to create a heat-dissipative thin copper oxide layer on metallic materials used for heat sinks. To maximize the heat dissipation of the oxidized copper layer, a copper layer with a rough surface was deposited by controlling the electroplating conditions, and the surface color, morphology, oxide thickness, thermal diffusivity, and emissivity were analyzed. In addition, we demonstrate the enhanced heat dissipation of the oxidized copper layer by applying the surface as a heat sink for a commercial LED module and measuring the operating temperature.

## 2. Materials and Methods

A mechanically polished copper plate (thickness: 0.2 mm, copper substrate (CS)) was cut into 40 mm × 25 mm pieces, which were used as substrates for surface modification. The substrates were cleaned in ethanol by ultrasonication for 5 min, degreased by dipping in 15 wt % NaOH for 200 s, and activated in 15 wt % HCl for 20 s. The samples were rinsed with deionized water for each treatment. The electrodeposition of copper was performed in 0.25 M CuSO_4_·5H_2_O + 0.5 M H_2_SO_4_ + 2.5 mM polyethylene glycol (PEG, average molar weight: 400) aqueous solution. The temperature of the electrolyte was maintained at 25 °C using a water-jacketed beaker with a chiller, and the electrolyte was stirred with a magnetic bar. For copper electrodeposition, a cathodic direct current of 50 mA/cm^2^ was applied to the copper substrate for 30 min. To form the copper oxide on the electrodeposited copper surface (EC), the sample was heat treated in an electric furnace at 300 °C for 10 min (HT10), 30 min (HT30), 50 min (HT50), and 70 min (HT70).

The surface morphologies and cross-sections of the samples were observed using field emission scanning electron microscopy (FE-SEM, Mira 3 LMH, Tescan, Brno, Czech Republic) with a focused ion beam (FIB, Helios G4 UC, Thermo Fisher Scientific, Waltham, MA, USA). Energy-dispersive X-ray spectroscopy (EDS) was also used for the chemical analysis of the surface metal and oxide layers. The crystal structure and composition of the oxides were studied using X-ray diffraction (XRD, SmartLab, Rigaku, Tokyo, Japan) in the angular range of 20–80°. The surface emissivity was measured using Fourier transform infrared spectroscopy (FT-IR, M4400-2-2S, MIDAC, Westfield, MA, USA) at 50 °C, and the thermal diffusivity through the surface layers was measured by laser flash analysis (LFA, LFA427, NETSZCH, Selb, Germany). To evaluate the heat dissipation, the surface-treated sample was attached to an LED module (RGB module 3.3V, Yahboom Co., Shenzhen, China) with thermal grease to remove air pockets. A thermocouple was inserted between the LED module and the heat sink, and the temperature was measured.

## 3. Results and Discussion

**Figure 1:**
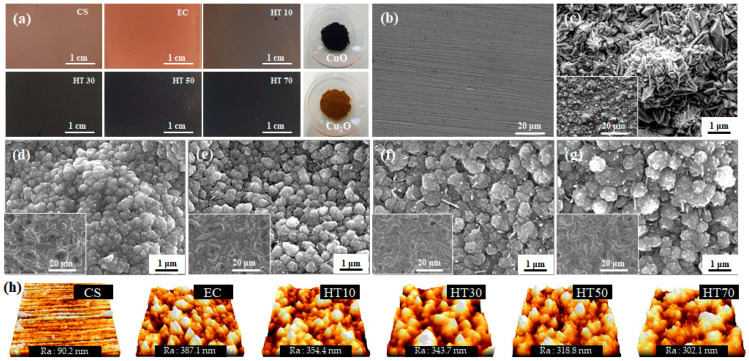
(**a**) Optical images of copper substrate (CS) and electrodeposited copper (EC) with heat treatment for 10 (HT10), 30 (HT30), 50 (HT50), and 70 (HT70) min. Surface SEM images of (**b**) CS, (**c**) EC, (**d**) HT10, (**e**) HT30, (**f**) HT50, and (**g**) HT70. (**h**) AFM images of sample surface and averaged roughness.

Because CuO and Cu_2_O have higher emissivity than Cu metal, the formation of copper oxides on the copper surface can be identified from the surface appearance. Figure 1 shows the optical and SEM images of the oxidized electrodeposited copper surfaces with various heat-treatment durations. The surface appearance of the copper plate, which exhibited polishing hairlines (Figure 1b), was changed by copper electrodeposition, which created a rough crystal structure (Figure 1c). In addition, the formation of a rough crystal structure significantly increased the surface roughness from ~90 nm to ~389 nm. Such a rough surface is expected to provide a larger effective area for thermal radiation. The heat treatment of the copper surface resulted in a more dramatic appearance change. In general, copper forms two surface oxides, namely brown Cu_2_O and black CuO [40]. As the heat-treatment duration increased, the surfaces became darker in color (Figure 1a), which indicates that oxides were formed during the heat treatment. Such change in the surface appearance to a darker color indicates an increase in the surface thermal emissivity [41]. In addition, the heat treatment significantly changed the surface morphology of the electrodeposited copper to form porous agglomerated nanospherical particles. This structure provides a more effective surface for thermal emission, enhancing the heat dissipation of the modified surface. The slight increase in the size of the spherical particles indicates the growth of copper oxides with an increase in the heat-treatment duration. During the heat treatment, the volume of the copper layer expands to become an oxide layer, thus the sharp crystal structure formed by electrodeposition collapses to become an agglomerated nanospherical particle structure. Therefore, with an increase in the heat-treatment duration, the surface roughness slightly decreases, such as 354 nm, 344 nm, 319 nm, and 302 nm for HT10, HT30, HT50, and HT70. The effect of the heat-treatment duration on the surface morphology was less significant than its effect on the appearance of the surface, which gained a darker color. In addition, the fact that the surface color of the heat-treated sample is much similar to the color of CuO suggests the formation of CuO at the top surface of the Cu layer.

**Figure 2:**
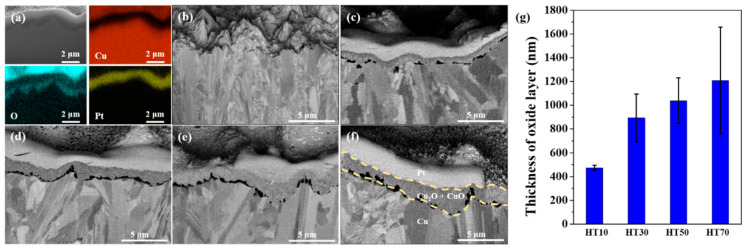
(**a**) EDS mapping of electrodeposited copper layer with heat treatment for 70 min (HT70). Cross-sectional SEM image of (**b**) electrodeposited copper layer (CS), and electrodeposited copper layer with heat treatment for (**c**) 10 min (HT10), (**d**) 30 min (HT30), (**e**) 50 min (HT50), and (**f**) 70 min (HT70). (**g**) Thickness of oxide layer formed by heat treatment.

Figure 2 shows a cross-sectional analysis of the heat-treated copper electrodeposited layer. Significant oxygen was detected on the top surface of the copper-deposited layer (Figure 2a), indicating the formation of copper oxides during the heat treatment. This oxide layer can be found in the cross-sectional images of the heat-treated samples (Figure 2c–f), while the oxide layer is absent from the electrodeposited copper surface (Figure 2b). The thickness of the oxide layer increased with heat-treatment duration from 470 ± 20 nm to 890 ± 200 nm, 1040 ± 190 nm, and 1210 ± 450 nm for HT10, HT30, HT50, and HT70, respectively. Since the formation of copper oxide depends on the transportation of oxygen to the copper/oxide interface through the oxide layer, the growth rate decreases with the thickness of the oxide layer. The growth of the oxide layer is more significant than the change in surface morphology caused by heat treatment, thus the surface color change is a better indicator of the thickness of the oxide layer.

**Figure 3:**
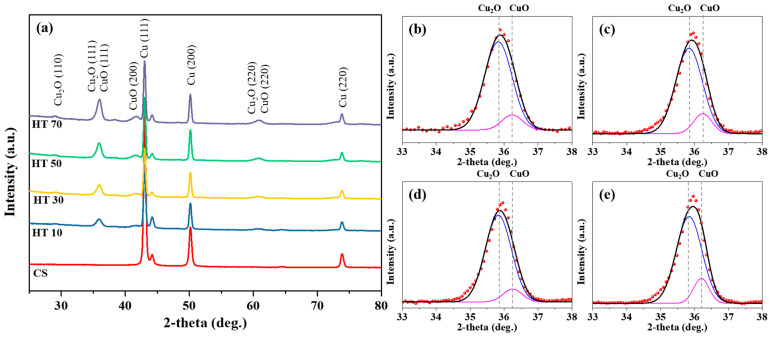
(**a**) X-ray diffraction of electrodeposited copper surface (CS) and electrodeposited copper surface with heat treatment for 10 min (HT10), 30 min (HT30), 50 min (HT50), and 70 min (HT70). Peak deconvolution (33–38°) to CuO (36.24°) and Cu2O (35.82°) for (**b**) HT10, (**c**) HT30, (**d**) HT50, and (**e**) HT70. Dots and solid lines in (**b**–**e**) indicate measured and fitted data, respectively.

The crystal structure of the oxide layer was analyzed using X-ray diffraction (Figure 3). The peaks of the (111) and (200) planes of metallic copper were observed at 43.3° and 50.47°, respectively. After heat treatment, the peaks of the (110) and (111) planes of Cu_2_O in the oxide layer could be observed at 29.57° and 35.82°, respectively. In addition, the peaks of the (111) and (200) planes of CuO in the oxide layer could be observed at 36.24° and 42.56°, respectively. These results indicate that the oxide layer was composed of Cu_2_O and CuO [42,43]. The overlapping (111) peaks of the Cu_2_O and CuO peaks at approximately 36° were deconvoluted, as shown in Figure 3b–e. The area ratios of the CuO (111)/Cu2O (111) peaks were 0.1138, 0.1331, 0.1364, and 0.1422 for the oxide layers of HT10, HT30, HT50, and HT70, respectively (Table 1). The increase in the area ratio of CuO (111)/Cu2O (111) with the heat-treatment duration suggests that the initial oxidation state of copper was Cu_2_O, and CuO was formed from Cu_2_O. In the initial stage of heat treatment, the copper surface was covered with Cu_2_O and had a brighter appearance than that of the copper surface covered with CuO. As the heat-treatment duration increased, the Cu_2_O surface was oxidized to CuO and gained a darker color. In addition, the diffusion of oxygen to the interface between the oxide layer and copper metal created more CuO in the oxide layer. For these reasons, as the heat-treatment duration increased, the surface of the electrodeposited copper layer gained a dark blackish appearance. In addition, the diffusion of oxygen through the oxide layer causes more Cu_2_O formation at the interface between the oxide layer and copper metal, thus the thickness of the oxide layer increases with the heat-treatment duration.

**Figure 4:**
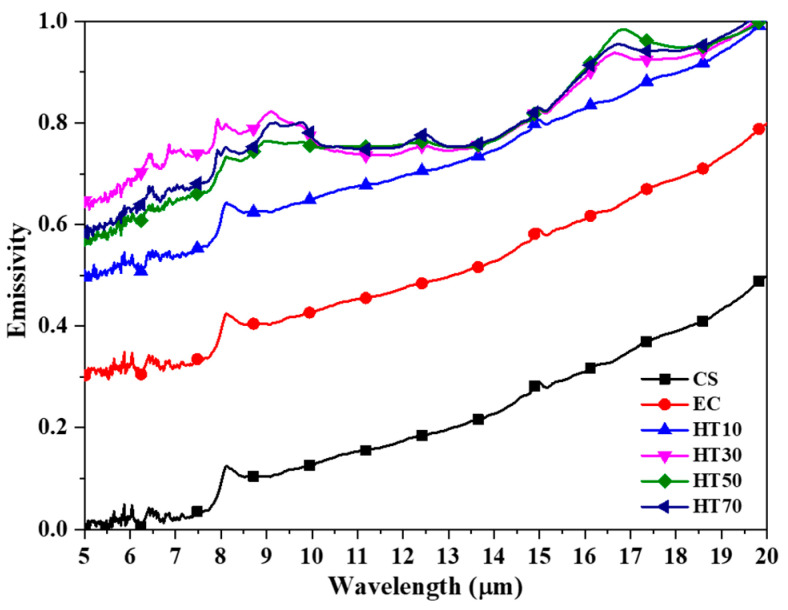
Emissivity of electrodeposited copper surface (CS) and electrodeposited copper surface (EC) with heat treatment for 10 min (HT10), 30 min (HT30), 50 min (HT50), and 70 min (HT70) in a wavelength range of 5–20 μm.

In general, a ceramic material with a dark color has a high emissivity of more than 0.8. The thermal emissivity of surface-treated copper was measured using Fourier transform infrared spectroscopy (FT-IR) in the wavelength range of 5–20 μm, and the results are shown in Figure 4. In addition, the average emissivity of each sample is summarized in Table 2. The emissivity of the metallic copper surface was increased from 0.279 (CS) to 0.579 (EC) by electrodeposition, which created a rough surface morphology of copper. This result indicates that the actual surface area for thermal radiation is important for controlling the surface emissivity. The surface emissivity was significantly increased by heat treatment. In addition, the emissivity increased with the duration of heat treatment from 0.794 to 0.857, 0.861, and 0.865 for HT10, HT30, HT50, and HT70, respectively. These results are in good agreement with the change in the surface appearance with the heat-treatment time. Therefore, surface modifications to achieve a darker appearance can improve thermal emissivity and enhance heat dissipation in the passive cooling state [44,45].

**Figure 5:**
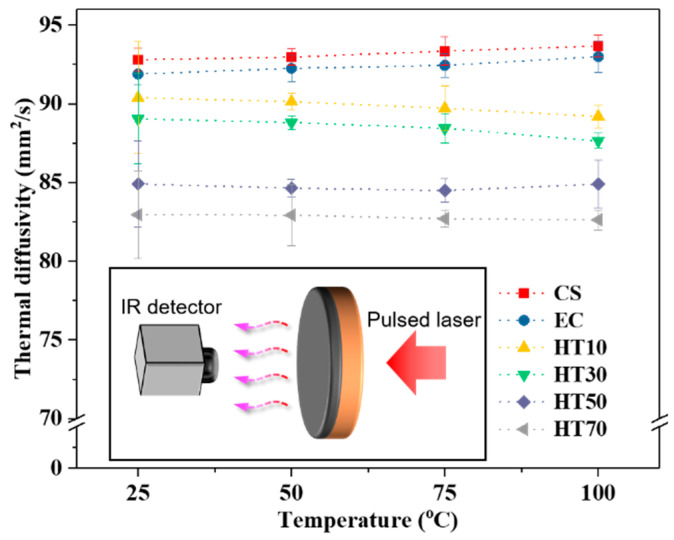
Thermal diffusivity of electrodeposited copper surface (CS) and electrodeposited copper surface (EC) with heat treatment for 10 min (HT10), 30 min (HT30), 50 min (HT50), and 70 min (HT70) measured at 25–100 °C.

The heat treatment resulted in the formation of CuO, which has a high emissivity, thereby enhancing the emissivity of the surface. However, CuO has a significantly lower thermal conductivity (~33 W/m∙K) than copper metal (~400 W/m∙K) by less than 8.3%. In addition, the CuO formed at the surface shows a nanospherical structure, which has a boundary between particles. Therefore, the actual thermal conductivity of the copper oxide layer would be lower than that of CuO, so the oxide layer can be a thermal barrier if only the thermal conduction through the oxide layer is considered [46]. This suggests that the increase in the oxide thickness also increases the thermal resistance between the copper substrate and the oxide surface. To compare the thermal resistance of the oxide layer, the thermal diffusivity of the sample was measured using LFA at various temperature ranges. We measured the thermal diffusivity five times for each sample and temperature. Averaged values with standard errors are shown in Figure 5. The laser source was irradiated on the opposite side of the oxide layer to generate heat, and the temperature was detected on the surface-treated side (i.e., oxide surface, inset in Figure 5). The thermal diffusivity was measured in the 25–100 °C range, but the thermal diffusivity of the tested sample did not show any significant variations with the testing temperature. The thermal diffusivity of CS was approximately 93 m^2^/s and decreased slightly to approximately 92 m^2^/s (EC) after electrodeposition, which created a rough surface morphology. The thermal diffusivity was significantly decreased as a result of the formation of an oxide layer. The thermal diffusivity of HT10 was approximately 90 m^2^/s, which is lower than that of EC. With an increase in the heat-treatment duration, the thermal diffusivity gradually decreased from approximately 88 m^2^/s for HT30 to 84 m^2^/s for HT50 and to approximately 82 m^2^/s for HT70. Such a decrease in thermal diffusivity with the heat-treatment time suggests that the heat transfer through the oxide layer was reduced. Such decreased heat transfer is attributed to the thickness increase of the oxide layer, which has much lower thermal conductivity than base copper metal. These results suggest that the optimization of heat-treatment duration to get an oxide layer with low thermal resistance and high emissivity is important for the best heat dissipation performance.

**Figure 6:**
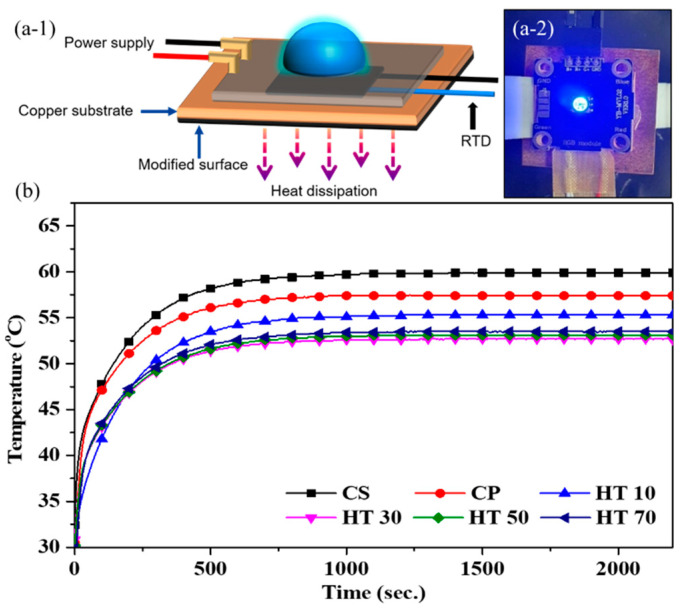
(**a-1**) Schematic and (**a-2**) photo of test setup with LED module and heat sink. (**b**) Temperature of LED module assembled to a heat sink with copper surface (CS) and electrodeposited copper surface (EC) with heat treatment for 10 min (HT10), 30 min (HT30), 50 min (HT50), and 70 min (HT70).

Although HT70 showed the highest emissivity, its thermal diffusivity was the lowest among the test samples. Therefore, the heat dissipation performance of surface-treated copper plates should be optimized by considering the test system and not just through material characterization. In this work, we prepared an LED module attached to a copper plate heat sink (Figure 6a). A resistance temperature detector (RTD) was then inserted between the LED module and the heat sink, and the temperature was recorded during the operation of the LED. The heat dissipation performance of the surface-treated copper heat sink was compared with the temperature of the operating LED. We tested each sample seven times, and representative temperature changes are shown in Figure 6b. After the operation of the LED, the temperature rapidly increased, and then the temperature stabilized at approximately 750 s. For quantitative comparison, seven stabilized temperatures for each sample were averaged. For CS, the temperature of the LED module was stabilized at 59.9 ± 1.0 ℃. For EC, which had a rough surface morphology, the stabilized temperature of the LED module was 57.5 ± 1.3 °C, which is lower than that of CS by 2.4 ℃. This enhanced heat dissipation was due to the increased surface area of the rough electrodeposited copper layer, which increased the thermal emissivity. The heat treatment of EC enhanced the heat dissipation, which further decreased the temperature of the LED module to 55.2 ± 0.3 ℃ for HT10, 52.6 ± 0.7 ℃ for HT30, 53.0 ± 0.6 ℃ for HT50, and 53.7 ± 0.5 ℃ for HT70. Ten minutes of heat treatment (HT10) forming an oxide layer with an emissivity of 0.794 enhanced the thermal radiation of the heat sink, thus the LED could be operated at a lower temperature by 2.3 ℃. Such an effect improving the heat dissipation is enhanced by the increase in heat-treatment duration to 30 min, which shows an emissivity of 0.857, thus the operating temperature for HT30 is lower than that of HT10. However, the operating temperature slightly increases with an increase in the heat-treatment time to more than 30 min, while the emissivity of HT50 (emissivity: 0.861) and HT70 (emissivity: 0.865) are slightly higher than that of HT30. These results are attributed to the thickness increase of the oxide layer, increasing the thermal resistance with the heat-treatment duration [47,48]. In order to enhance the heat dissipation, the surface modification to realize a high emissivity without the formation of a thermally resistant layer would be desirable for improving heat dissipation in passive cooling. However, the heat treatment of copper forming the blackish oxide layer causes opposite effects on the heat dissipation, enhancing thermal radiation with emissivity and increasing thermal resistance through the oxide layer. For these reasons, in this study, the heat treatment of the copper electrodeposited layer for 30 min shows the most effective heat dissipation. In addition, it should be noted that if the surface modification for enhancing the emissivity forms a thermally resistant layer, optimization is required for maximizing heat dissipation.

The electrodeposition of copper used in this study is a widely used process for copper and aluminum heat sinks and can be applied to complex shapes. In addition, heat treatment to form an oxide layer on copper can be performed in a general electric furnace or oven. Therefore, the method demonstrated in this work to enhance the heat dissipation of heat sinks in the passive cooling state can solve various heat management problems and can be directly applied to practical fields.

## 4. Conclusions

The heat treatment of the electrodeposited copper surface creates a rough oxide layer containing CuO and Cu_2_O, which have high thermal emissivity. With the increase in the heat treatment duration, the surface gained a darker appearance, and the thickness of the oxide layer increased, resulting in the enhancement of thermal emissivity. Since the emissivity improves the thermal radiation, the enhanced thermal emissivity of the heat sink surface for the LED module contributed to the decrease in the LED operating temperature, indicating an improvement in heat dissipation in the passive cooling state. However, the thermal conductivities of copper oxides were significantly lower than copper metal, and the thermal diffusivity through the oxide layer decreased with an increase in the heat treatment time, which increased the thermal resistance in the heat transfer path. Insufficient heat treatment of the copper surface causing lower emissivity results in low thermal radiative heat dissipation, while excessive heat treatment creates a too-thick thermally resistive oxide layer. Therefore, considering heat transfer in passive cooling, the heat treatment of the copper surface shows not only a positive effect of enhancing thermal radiation but also a negative effect of increasing thermal resistance. These results suggest that the best heat dissipation performance of the heat sink can be achieved by optimization of the surface thermal emissivity and thickness of the oxide layer.

## Figures and Tables

**Table 1:** Calculated CuO/Cu_2_O ratios of electrodeposited copper surface with heat treatment for 10 min (HT10), 30 min (HT30), 50 min (HT50), and 70 min (HT70).

	HT10	HT30	HT50	HT70
CuO/Cu_2_O	0.1138	0.1331	0.1364	0.1422

**Table 2:** Averaged emissivity of electrodeposited copper surface (CS) and electrodeposited copper surface (EC) with heat treatment for 10 min (HT10), 30 min (HT30), 50 min (HT50), and 70 min (HT70) in a wavelength range of 5–20 μm.

	CS	EC	HT10	HT30	HT50	HT70
Emissivity	0.279	0.579	0.794	0.857	0.861	0.865

## Data Availability

Not applicable.
